# Regular Physical Activity as a Potential Risk Factor for Erosive Lesions in Adolescents

**DOI:** 10.3390/ijerph17093002

**Published:** 2020-04-26

**Authors:** Kacper Nijakowski, Anna Walerczyk-Sas, Anna Surdacka

**Affiliations:** 1 Department of Conservative Dentistry and Endodontics, Poznan University of Medical Sciences, 60-812 Poznań, Poland; 2 Private Dental Practice, 62-020 Zalasewo, Poland

**Keywords:** erosive lesion, physical activity, adolescents

## Abstract

Tooth erosion is becoming an increasingly common dental problem among teenagers. The study aimed to determine risk factors for erosive lesions in young sports professionals. Participants were 155 students—102 physically active and 53 controls. The method included dental examination (including Basic Erosive Wear Examination) and a questionnaire concerning sports activity, dietary and hygienic habits. The sporting activity significantly correlated with erosive lesions (R_Spearman_ = 0.344). The regression model incorporating the kind of sports activity, special diet and (non-)drinking water was statistically significant (*p* = 0.922 for the Hosmer–Lemeshow test) and strong enough to predict erosive lesions in young athletes (AUC = 0.758). Water sports professionals were almost 14 times more likely to suffer from erosive lesions than control students. Drinking water as the main beverage decreased the odds by about 70%. The graphical interpretation of multidimensional correspondence analysis confirms the predictive value of these factors. The kind of sports activity adjusted by the main beverage and a special diet was the best variable to predict erosive lesions among teenagers. Early proper diagnosis with defined risk factors leads to better prevention and successful treatment.

## 1. Introduction

**Figure 1:**
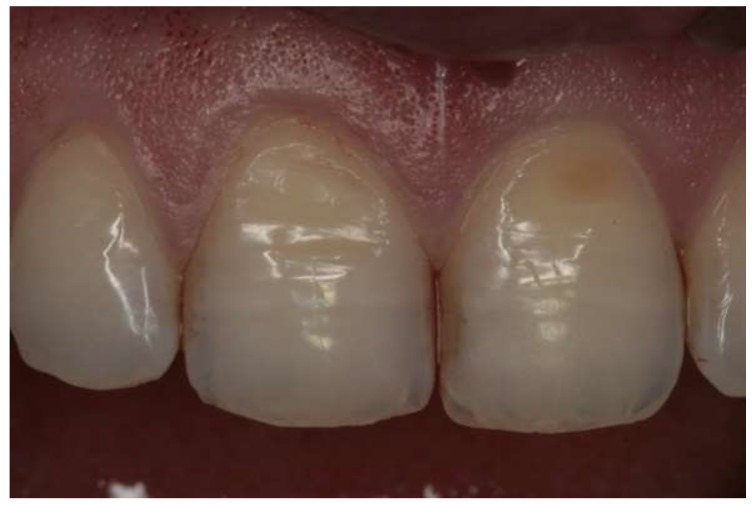
Erosive lesions on labial surfaces of central incisors (personal collection).

**Figure 2:**
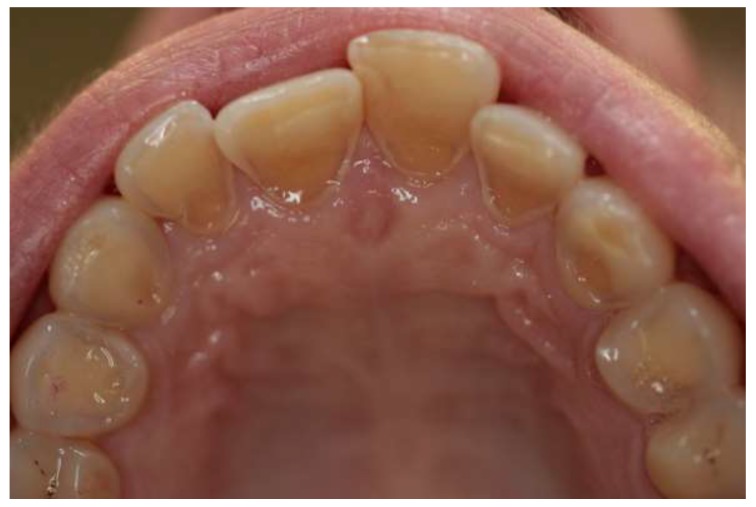
Erosive lesions on palatal surfaces of incisors and canines (personal collection).

Erosive lesions are caused by acids of nonbacterial origin, e.g., the citric acid contained in the diet, the hydrochloric acid of refluxing stomach contents in gastric disorders. Tooth erosion is characterized by shallow, fairly extensive cavities with a preserved enamel on the gingival margin [1,2,3]. Despite a specific clinical picture (Figure 1 and Figure 2), it is often difficult to establish a proper diagnosis. Many researchers emphasize the importance of the interaction of etiological factors responsible for the process of noncarious hard tissue loss. It is essential to conduct a detailed interview to make an accurate diagnosis and to implement appropriate preventive and therapeutic methods.

Erosion prevention is based on limiting the frequency of consuming low pH products. Patients should receive oral hygiene instructions such as tooth brushing at least one hour after consumption of products with high acidity and using low abrasive pastes. In the literature, it is recommended to apply fluoride-free preparations directly on the surface of teeth, e.g., Tooth Mousse GC (containing casein phosphopeptides with amorphous calcium phosphate – CPP-ACP). The effectiveness of the CPP-ACP complex in the prevention of erosion caused by acidic isotonic drinks has been repeatedly described [4,5,6]. The selection of fluoride prophylaxis should take into account the concentration of fluorine in drinking water in the area. In addition to professional dental care, it is necessary to conduct appropriate prohealth education in primary and secondary schools.

The diet of a young active person is often rich in products with high erosive potential. In a study among students practicing sports, Ostrowska et al. [7] found that erosions occurred in 31.4% of the respondents, of which 68.1% consumed isotonic drinks frequently (several times a week or more often) and 31.9% rarely (once a week or even less). Mulic et al. [8] draw attention to the lowered secretion of saliva during physical exercise and its supposed association with the formation of erosion among athletes. The decrease in saliva secretion during intense physical effort and the consumption of isotonic drinks qualify athletes to be part of the group with increased risk of erosion [9,10,11].

The results of Kaczmarek’s research [12], conducted among professionals practicing in chlorinated water pools, show the location of erosive lesions limited mainly to the labial surfaces of the maxillary incisors. The author explained that swimmers are more likely to develop erosions of tooth enamel than people who have not practiced this sport. In this study, erosive lesions were more frequent in men than in women. Milosevic et al. [13] demonstrated the occurrence of erosion in swimmers, especially on the labial and palatal surfaces of upper incisors. In the lateral teeth, the authors observed erosive lesions on buccal and palatal surfaces in the maxilla, as well as buccal and occlusal surfaces in the mandible.

Based on the above reports, it is assumed that young physically active people are more likely to develop erosive lesions than inactive people. It should be stressed that there are few publications in the literature on the prevalence of tooth erosion in sports professionals. Our study aimed to assess the influence of sports activity on erosive lesions in adolescents, especially to determine potential risk factors, considering kind of sport, dietary and hygienic habits, salivary flow rate and gender.

## 2. Materials and Methods 

The study involved a total of 155 people (both genders), from secondary school (Athletic Championship High School in Poznan). Students were drawn to the research group from classes with a sports profile, and to the control group from the others, not declaring regular physical activity. The selection criteria were only—age (between 15 and 18 years of age) and doing sports.

The clinical examination was carried out in the offices of school hygienists, in conditions of artificial lighting, with all aseptic principles. The BEWE index (Basic Erosive Wear Examination index), developed by an international team of experts [14], was used to assess the risk of tooth erosion.

This index allows us to determine the distribution and severity of erosive lesions in the dentition, regardless of their origin. Due to its criteria, it is easy to use and can be used both in clinical practice and in research. Dentition is divided into sextants, and each tooth in a given sextant is examined for the occurrence of erosion signs and then erosive lesions are classified on a 4-grade scale:

Grade 0: no erosive changes;
Grade 1: initial loss of enamel texture;
Grade 2: visible loss of hard tissues under 50% of the surface; and
Grade 3: visible loss of hard tissues over 50% of the surface.

The BEWE index is obtained by summing up the highest recorded values for each sextant. The total value determines the severity of tooth erosion and the risk of erosive lesions occurring in the future:

BEWE 0-2: no risk (code 0);
BEWE 3-8: low risk (code 1);
BEWE 9-13: average risk (code 2); and
BEWE ≥14: high risk (code 3).

The assessment of the potential risk factors for erosive lesions was carried out by means of a survey on sports activity, general health, dietary and hygienic habits. The questionnaires were completed by the patients in cooperation with the doctor immediately before the examination. Moreover, the stimulated saliva was collected for a total of 10 min and the secreted amount was calculated in mL/min.

The interval variables were compared using the Mann–Whitney’s test (not normally distributed in the Shapiro–Wilk test) and nominal variables using the Pearson’s Chi^2^ test. Correlations were determined using the Spearman’s rank test. The advanced statistical methods (i.e., logistic regression modelling and multidimensional correspondence analysis) were used to assess the relationship between potential risk factors and the prevalence of erosive lesions. The significance level was estimated at α = 0.05. The analyses were performed using the Statistica 13.3 software (StatSoft, Cracow, Poland).

This study was approved by the Bioethical Commission at Poznan University of Medical Sciences (No. 1147/12). Every patient was informed about the aim and type of research to be carried out and signed written consents for participation were obtained from all patients and their parents. All procedures performed in studies involving human participants were in accordance with the 1964 Helsinki declaration and its later amendments or comparable ethical standards.

## 3. Results

### 3.1. Basic Statistics

The distribution of students by kind of sports activity is shown in Table 1.

The summary table of quantities (Table 2) presents the percentage of students exposed to tooth erosion depending on the selected potential risk factor, i.e., type of sport, diet and hygiene habits (including *p*-values of the Pearson’s Chi^2^ test). The differences between the amount of stimulated saliva were not statistically significant (medians in both groups equal 1 mL/min; *p*-value = 0.080).

To confirm the influence of sports activity on the presence of erosive lesions in adolescents, a significant correlation between sport and the risk of erosion was demonstrated (Spearman correlation coefficient = 0.344).

### 3.2. Logistic Regression Model

In order to better explain the relationship, a logistic regression model was constructed, which, apart from the type of sport, takes into account predictors with the highest values of d-Somers coefficient, significantly improving its quality. These include a special diet and a mainly consumed beverage (in this case, water with the highest predictive value is included to the model). Table 3 shows the parameters of the predictors incorporated in the model. The calculated odds ratios indicate that the selected types of sports increase the chance of erosive defects in relation to the control group not practicing any sports—especially water sports (including swimming) as many as 14 times. Moreover, consumption of mainly water reduces the odds by 70% and the special diet of athletes increases almost four times.

**Figure 3:**
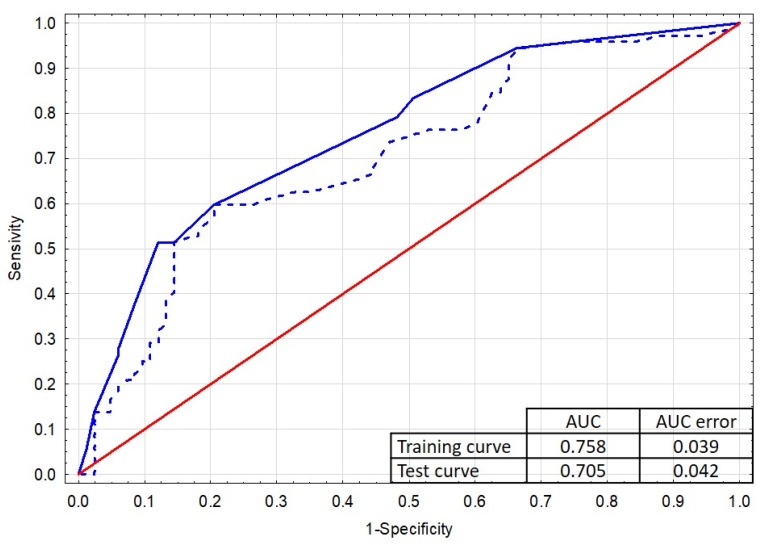
V-fold cross validation—learning ROC curves. Blue solid line—training curve, blue dashed line—test curve, red line—reference line.

The Hosmer–Lemeshow test was used to assess the goodness of fit, whose high *p*-value = 0.922 does not allow to reject the zero hypothesis of the equality of observed and predicted values—the model is well-fitted to the data. The model also has a relatively low AIC (Akaike Information Criterion = 192.4). In addition, the model was validated by the v-fold cross method and the ROC (receiver operating characteristic) curves (Figure 3) for the training and testing sample were obtained. The high value of the area under the curve for the training curve (AUC = 0.758) and the small difference in areas between the superimposed curves of both samples (<0.1) confirm the good quality of the model.

### 3.3. Multidimensional Correspondence Analysis

**Figure 4:**
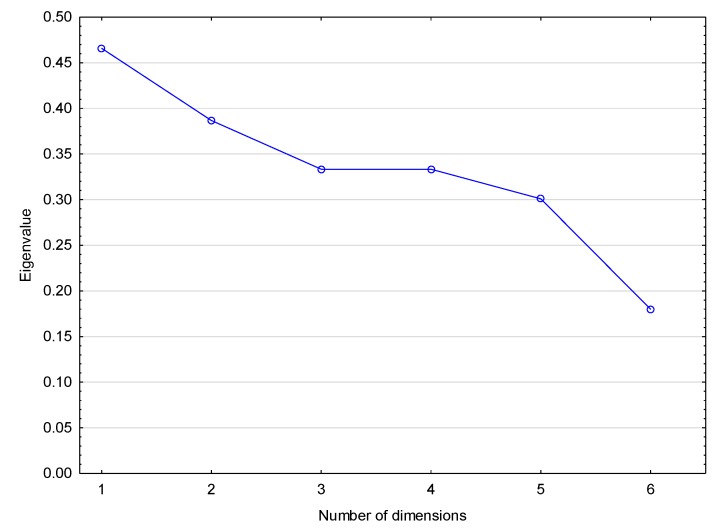
Plot for multidimensional correspondence analysis.

**Figure 5:**
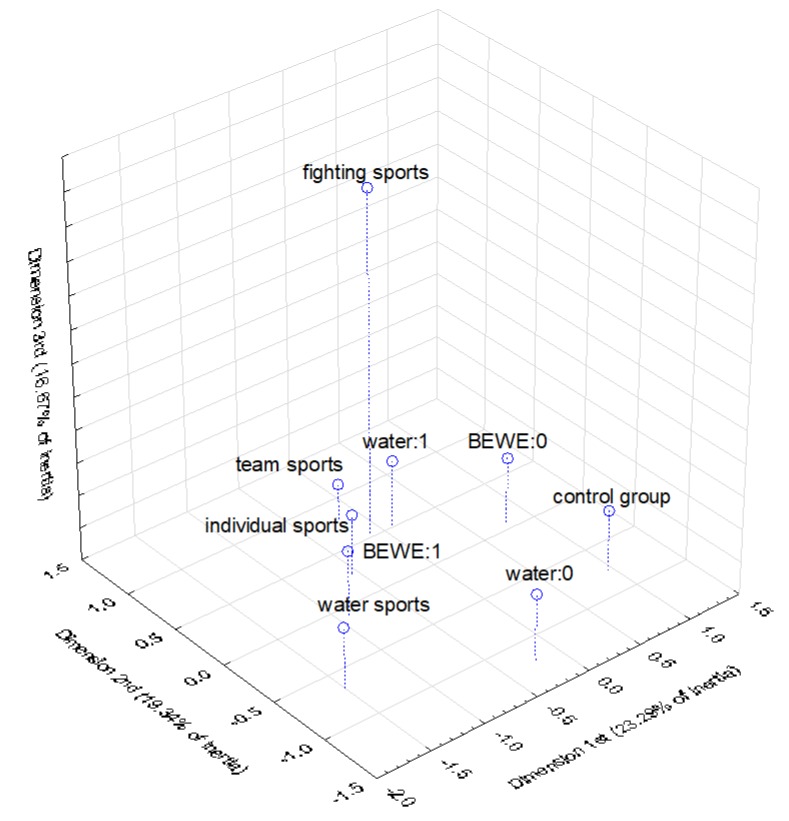
Multidimensional correspondence analysis—3-dimensional plot.

**Figure 6:**
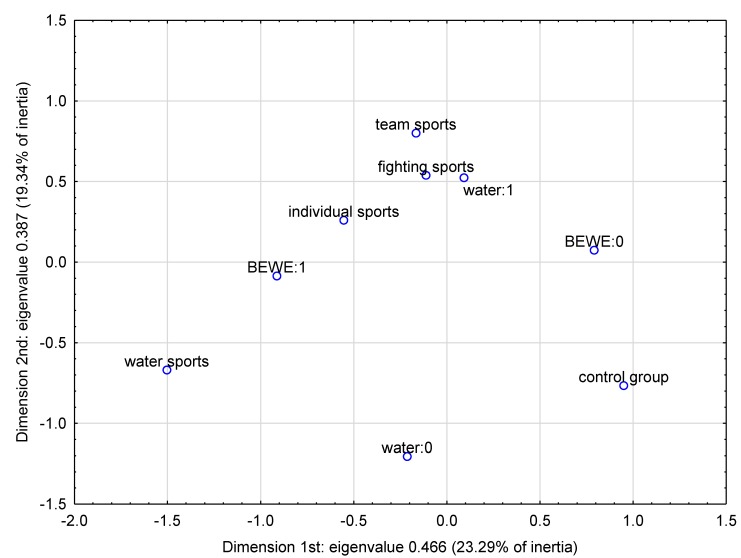
Multidimensional correspondence analysis—2-dimensional plot.

In order to graphically illustrate the relationship between the relevant model factors and the occurrence of erosion, a multidimensional correspondence analysis (according to Burt’s table 9 × 9) was conducted. On the basis of the scree plot (Figure 4), it was decided to choose three-dimensional analysis as the best description of the examined phenomenon. Figure 5 and Figure 6 show the results of the analysis in three dimensions, as well as in two dimensions. Table 4 contains parameters characterizing the determined points. In the case of the three-dimensional plot, the points representing water sports and individual sports as well as nonwater consumption are concentrated closest to BEWE = 1, which indicates a higher probability of erosion development for these factors. The two-dimensional plot confirms the strongest relationship between erosion risk and water sports (the smallest angle with vertex at the beginning of the coordinate system).

## 4. Discussion

Our study has identified potential risk factors for erosive lesions in young athletes, such as kind of sport (especially water sports) and consumption of isotonic drinks. All students who presented any risk factor were informed about prevention and treatment methods.

A clinical examination using the BEWE index showed the occurrence of erosive lesions in 59.80% of all athletes. The control group was less affected (22.64%). The differences were statistically significant (*p*-value < 0.0001). The obtained values of the BEWE index indicate a low risk of erosion among the examined group of adolescents.

Based on the results of the study conducted in 2014 as part of the Oral Health Monitoring, Strużycka et al. [15] observed tooth erosion in 42% of 18-year-olds. The same research reported a low risk of erosion in 28.9% (BEWE 1) and more advanced lesions occupying up to 50% of the tooth surface in 11.9% of all individuals (BEWE 2). Advanced lesions occurred very rarely and concerned only 1.5% of the examined population (BEWE 3).

The analysis of dietary and hygienic habits has repeatedly shown that adolescents are a group particularly exposed to the formation and development of erosive lesions. Foreign authors also stress the increasing tendency of the occurrence of the noncarious defects in these patients. The study among 18-year-olds from Oslo in 2012 showed that 54% of the respondents were affected by tooth erosion [16]. The research conducted in Sweden reported a higher percentage of erosive lesions among young adults of up to 75% [17]. Moreover, Margaritis et al. [18] evaluated the erosion process in 14-16-year-old Greek adolescents on the basis of the BEWE index—its incidence was 58%.

In the examined group of athletes, erosive lesions most often occurred in the mandible and included the fourth sextant (68.85%) and sixth sextant (65.57%), as well as the second sextant (55.73%) in the maxilla. Results obtained by Bachanek et al. [19] indicate the most frequent occurrence of erosion among the surveyed group of young adults was in the second (27.01%), fourth and sixth (22.63%) sextants. Kaczmarek et al. [20], in their research conducted among 15-year-old adolescents from Opole province, draw attention to the symmetric distribution of lesions in the lateral sextants of the maxilla and mandible. The lesions in the maxilla were equally frequent but in the mandible significantly more often in lateral sextants than in the anterior one. A study conducted by the same author among patients exposed to endo- and exogenous acids showed that the highest intensity of lesions occurred in second and fifth sextants, within anterior teeth [21]. In our research, a similar distribution was obtained.

Special attention should be paid to young people who practice swimming. Already in 1997, Milosevic et al. [13] suggested a presumed link between the occurrence of erosion and the consumption of sour drinks among athletes. In the group of swimmers and cyclists, they diagnosed erosion in 36% of respondents. In swimmers, Geurtsen [22] describes erosive lesions located on the labial surfaces of the maxillary incisors. Furthermore, in 2010, Kaczmarek [12] conducted the aforementioned earlier research among the population of young swimmers. The author explains that dissolved chlorine forms compounds in water that most likely chelate mineralized tooth tissues acting on them as food acids. The study carried out on the group of adolescents aged 14–16 years, who swim professionally, and the group of adolescents of the same age, who swim recreationally, showed a statistically significant relationship (*p*-value = 0.008) between professional swimming in chlorinated water and the risk of erosion on the labial surfaces of the upper central incisors. Erosive lesions were found in over 26% of professional swimmers and 10% of recreational swimmers (*p*-value = 0.02).

Moreover, dietary factors play an essential role in the etiology of tooth erosion. The consumption of acidic foods leads to demineralization and dissolution of enamel hydroxyapatite crystals. Sports professionals take special care of their health and attach great importance to proper nutrition. They strictly follow the indications of dieticians, providing the body with appropriate amounts of vitamins and microelements. A survey on dietary habits conducted by Waszkiel [23] proved that there is a directly proportional relationship between the prevalence of erosion and the frequency of consumption of apples, fruit and citrus juices and carbonated drinks. Also, Al- Dlaigan et al. [24] have noted a link between the frequent consumption of fresh fruit and the presence of erosion defects. They found that more than 80% of 14-year-old children in England drank soft drinks every day. As was emphasized by Waszkiel [23], it is currently believed that the frequency of acid consumption (not the amount) determines the formation of tooth erosion. A single ingestion of even a significant amount of low pH foods will be neutralized in a short time by saliva. Moreover, Søvik et al. [25] revealed a significant association between dental erosion and high consumption of sport drinks (0.75–5 L/day) among adolescents. In contrast, Antunes et al. [26] have not shown any significant relationship for isotonic drinks in amateur runners but suggested frequency of exercise per week as a potential risk factor.

In own research, drinking water as the main beverage is declared by only about 1/3 of respondents, and about half of them consume fresh fruit more than twice a week. According to Prymas et al. [27], acidic fruit most often causes erosive lesions on the front teeth and fruit juices or drinks with low pH on the lateral teeth. However, some authors point out that erosion is not linked to fruit consumption [28]. Nutritional recommendations, especially for people who care about their health and physical condition, indicate a minimum of five times a day consumption of fruit and vegetables. They are a rich source of vitamin C and microelements necessary for the proper functioning of the body. Respecting dietary recommendations, a comprehensive action should be taken to inform patients about the principles of protecting their teeth from the harmful effects of low pH foods.

Mulic et al. [16] examined people practicing sports aged 18-35 and assessed the effect of physical exercise on the minute output of stimulated saliva. They showed a significant relationship between the occurrence of erosion in athletes with reduced flow of stimulated saliva (*p*-value < 0.01). The mean value of saliva after stimulation before exercise was 1.43 mL/min and after exercise 1.31 mL/min. The results were slightly higher than the values obtained in their own study (1.12 mL/min). Horswill et al. [29] demonstrated a statistically significant lower level of stimulated saliva secretion in sports professionals despite water consumption during training. A similar observation was confirmed by Frese et al. [30].

## 5. Conclusions

The influence of sport on the condition of hard dental tissues is not fully proven. Young sports professionals often follow a diet rich in fresh fruit and vegetables and low pH drinks. Physical effort can also affect the physicochemical properties of saliva. Therefore, young athletes can be classified as a group at risk of tooth erosion.

## Figures and Tables

**Table 1:** Distribution of students by type of sport.

	Female	Male	All
team sports	28	30	58
fighting sports	1	7	8
water sports	7	14	21
individual sports	12	3	15
control group	35	18	53
	83	72	155

**Table 2:** Selected potential risk factors for erosive lesions.

		BEWE > 0 [%]	*p*-Value
kind of sports activity	team sports	51.72	**<0.001**
	fighting sports	50.00	
	water sports	80.95	
	individual sports	60.00	
	control group	22.64	
main beverage	water	41.67	0.070
	isotonic drinks	80.95	**0.015**
	fruit juices	45.33	0.103
	carbonated drinks	53.85	0.732
	tendency to hold the drink in the mouth	57.14	0.136
brushing method	horizontal	50.00	0.401
	circular	45.21	
	sweeping	20.00	
	gastric disorders	77.78	0.052
gender	female	37.35	**0.015**
	male	56.94	

Bold for statistical significance *p*-value <0.05, according to the Pearson’s Chi^2^ test.

**Table 3:** Parameters of predictors incorporated into the logistic regression model.

	β	SE	Wald Stat.	*p*-Value	Odds Ratio	Confidence OR -95%	Confidence OR 95%
intercept	−0.616	0.388	2.520	0.112	0.540	0.252	1.155
team sports	1.418	0.462	9.423	**0.002**	4.129	1.670	10.209
individual sports	1.908	0.655	8.489	**0.004**	6.736	1.867	24.306
fighting sports	1.329	0.824	2.604	0.107	3.778	0.752	18.982
water sports	2.636	0.682	14.921	**<0.001**	13.957	3.664	53.171
special diet: 1	1.326	0.719	3.400	0.065	3.766	0.920	15.415
water: 1	−1.160	0.420	7.633	**0.006**	0.314	0.138	0.714

Bold for statistical significance *p*-value <0.05.

**Table 4:** Multidimensional correspondence analysis—parameters of determined points.

	x	y	z	Quality	Relative Inertia	x		y		z	
						Inertia	cos^2	Inertia	cos^2	Inertia	cos^2
team sports	−0.166	0.801	−0.467	0.530	0.104	0.007	0.016	0.207	0.383	0.082	0.131
individual sports	−0.553	0.259	−0.090	0.041	0.151	0.021	0.033	0.006	0.007	0.001	0.001
fighting sports	−0.111	0.539	4.211	0.982	0.158	0.001	0.001	0.013	0.016	0.915	0.965
water sports	−1.503	−0.669	−0.068	0.425	0.144	0.219	0.354	0.052	0.070	0.001	0.001
control group	0.950	−0.766	−0.072	0.777	0.110	0.221	0.469	0.173	0.305	0.002	0.003
BEWE: 0	0.792	0.074	0.000	0.729	0.077	0.240	0.723	0.003	0.006	0.000	0.000
BEWE: 1	−0.913	−0.086	−0.000	0.729	0.089	0.277	0.723	0.003	0.006	0.000	0.000
water: 0	−0.212	−1.204	−0.000	0.651	0.116	0.010	0.020	0.379	0.631	0.000	0.000
water: 1	0.092	0.524	0.000	0.651	0.051	0.004	0.020	0.165	0.631	0.000	0.000
